# Comparison of extended segmentectomy with traditional segmentectomy for stage I lung cancer

**DOI:** 10.1186/s13019-022-01771-4

**Published:** 2022-03-04

**Authors:** Ya-Fu Cheng, Yueh-Che Hsieh, Yu-Jun Chang, Ching-Yuan Cheng, Chang-Lun Huang, Wei-Heng Hung, Bing-Yen Wang

**Affiliations:** 1 Division of Thoracic Surgery, Department of Surgery, Changhua Christian Hospital, Changhua County, No. 135 Nanxiao St., Changhua City, 500 Taiwan, ROC; 2 Department of Emergency Medicine, Chang Gung Memorial Hospital, Keelung, Chang Gung University College of Medicine, Taoyuan, Taiwan, ROC; 3 Big Data Center, Epidemiology and Biostatistics Center, Changhua Christian Hospital, Changhua, Taiwan, ROC; 4 School of Medicine, Chung Shan Medical University, Taichung, Taiwan, ROC; 5 School of Medicine, College of Medicine, Kaohsiung Medical University, Kaohsiung, Taiwan, ROC; 6 Institute of Genomics and Bioinformatics, National Chung Hsing University, Taichung, Taiwan, ROC; 7 Department of Post-Baccalaureate Medicine, College of Medicine, National Chung Hsing University, Taichung, Taiwan, ROC; 8 Center for General Education, Ming Dao University, Changhua, Taiwan, ROC

**Keywords:** Extended segmentectomy, Image-guided video-assisted thoracoscopic surgery (iVATS), Resection margin, Non-small cell lung cancer (NSCLC)

## Abstract

**Background:**

For stage I non-small cell lung cancer (NSCLC), lobectomy and segmentectomy are still controversial operations. Extended segmentectomy was proposed to make larger safe margins than segmentectomy. Image-guided video-assisted thoracoscopic surgery (iVATS) is useful to accomplish extended segmentectomy. We aimed to compare the effects of iVATS extended segmentectomy to the effects of traditional segmentectomy for stage I NSCLC.

**Methods:**

This study is a retrospective analysis in a single institute. Patients with stage I NSCLC who received segmentectomy between January 2017 and September 2020 were included. Patients were distributed to iVATS extended segmentectomy (group A), and traditional segmentectomy (group B). The impacts of the different surgical methods on resection margin were assessed.

**Results:**

There were 116 patients enrolled in this study. Sixty-two patients distributed in group A, and the other 54 patients in group B. The resection margin to a staple line was 17.94 mm in group A versus 14.15 mm in group B, *p* = 0.037. The margin/tumor diameter ratio was 2.08 in group A versus 1.39 in group B, *p* = 0.003. The enough margin rate was 75.81% and 57.41%, respectively, for group A and group B. The subgroup analysis of iVATS extended segmentectomy showed that T1a lesions had larger margin distances than did T1b lesions (19.85 mm vs. 14.83 mm, *p* = 0.026).

**Conclusions:**

The iVATS extended segmentectomy can provide more resection margin than traditional segmentectomy. Segmentectomy is more suitable to perform when the nodule’s diameter is less than 10 mm.

## Background

Lung cancer has been the leading cause of cancer deaths worldwide over the past few decades [1]. As a result of advancements in cancer screening, more and more small pulmonary nodules are detected. For stage I non-small cell lung cancer (NSCLC), lobectomy and segmentectomy are still controversial operations. Lobectomy is criticized for much pulmonary function loss [2]. There were some studies showing that the overall survival is similar for the two operations when the tumor is less than 20 mm in diameter [3, 4]. However, questions have been raised as to whether segmentectomy has enough resection margin.

From the National Comprehensive Cancer Network (NCCN) guidelines, the term “enough resection margin” is classified as the margin length being at least 2 cm or the tumor’s size [5]. To achieve the criterion, extended segmentectomy was proposed to make larger safe margins [6, 7]. However, there was no precise method for extended segmentectomy. We describe a novel technique using image-guided video-assisted thoracoscopic surgery (iVATS) to accomplish extended segmentectomy. There were several studies that discussed the details of iVATS with cone-beam CT [8–10].

In this study, we aimed to compare the effects of iVATS extended segmentectomy and the effects of traditional segmentectomy for stage I NSCLC. We designed a retrospective study to find out if iVATS extended segmentectomy can create more resection margin than traditional segmentectomy.

## Methods

### Patient population and selection

This study is a retrospective analysis in our institute (Changhua Christian Hospital, Changhua, Taiwan). All patients over 18 years of age undergoing segmentectomy from January 2017 to September 2020 were included in the study. Patients without NSCLC or with positive lymph node invasion were excluded. Other exclusion criteria were the tumor being larger than 20 mm in diameter and missing pulmonary function data.

Our study was approved by the institutional review board in our institution (IRB-201004), and informed consent from all participants was waived. We analyzed the age, gender, tumor location, forced expiratory volume in one second (FEV1), tumor differentiation, tumor histology, pathologic T stage, tumor subtype, spread through air spaces (STAS), pleural invasion and lymphovascular invasion. We classified histologic subtypes as adenocarcinoma, squamous cell carcinoma, and other histologic types. Patients were divided into two groups: group A (received iVATS extended segmentectomy) and group B (received traditional segmentectomy). The decision to perform iVATS extended segmentectomy or traditional segmentectomy was made by surgeon’s preference. If the nodule was located at intersegmental area and hard to make enough resection margin, iVATS extended segmentectomy would be preferred. Otherwise, traditional segmentectomy would be performed if the nodule was in the central of segment.

The outcome measures for our study were the closest margin to a staple line, the margin/tumor diameter ratio (M/T ratio) and the enough margin rate. The chosen classification of margin was deflated lung margin based on the pathology reported by a qualified pathologist. The definition of enough margin was based on National Comprehensive Cancer Network (NCCN) practice guidelines, which described “sublobar resection should achieve parenchymal resection margins ≧ 2 cm or ≧ the size of the nodule.” [5] Every observation was staged according to the eighth edition of the TNM staging system, published in 2017 [11].

### Measurements and variables

Under general anesthesia, patients were positioned in the lateral decubitus position in a hybrid operating room. All lines and tubes were secured and taped. We used robotic C-arm cone beam CT (Artis Pheno; Siemens Healthcare GmbH, Forchheim, Germany) for the scanning. A test-C-arm-movement was performed to ensure the scanner would not collide with the patient before the scanning. The whole scan was performed with breath hold at end inspiration by clamping the endotracheal tube.

We measured the insertion point at axial view and laid out the needle path under the syngo Needle Guidance of a syngo X-Workplace with a three-dimensional view. The guidance needle was inserted to around a 10 mm depth in the pleura toward the nodule. A cross laser beam for incision location projected onto the patient’s skin. We punctured an 18-gauge marker needle into the thorax with the cross-laser guidance after holding. After another scan for confirmation of the appropriate needle location, diluted methylene blue dye (0.15 ml) plus normal saline (0.25 ml) were injected. The purpose of this mixture was for the methylene blue dye to be seen within 5 mm of the surface without coloring the nodule. The operation started after sterilization and one lung ventilation.

**Fig. 1:**
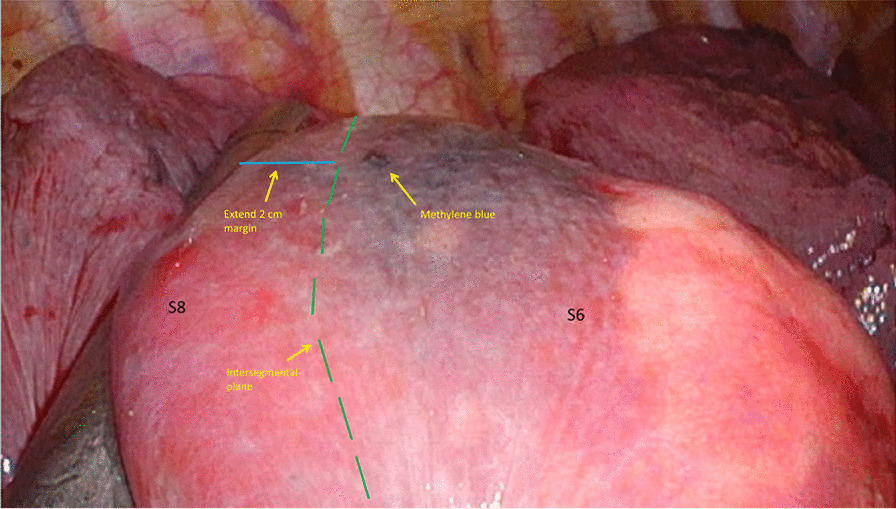
The nodule site and margin during an iVATS extended segmentectomy

We performed extended segmentectomy with single-incision VATS. Most of the methylene blue dye could be seen clearly on the surface of the lung. After the division of the segmental bronchus, we identified the inflation-deflation line and the nodule site. A safe margin of 2 cm away from the nodule site was created and divided with a linear stapler (Fig. 1).

On the other hand, we performed traditional segmentectomy while the nodule was clearly away from inter-segmental area. Pre-operative CT-guided micro-coil localization would be done one day before the operation in order to help finding the nodule intra-operatively. We also use the inflation-deflation method to identify the inter-segmental plane.

We routinely check resection margin immediately on a back table. We make sure the free margin and measure the resection margin on every pathology. If the margin is less than 5 mm, we would perform additional wedge resection to create more resection margin. We do not perform lobectomy in these patients due to the small nodules size and we tend to preserve more pulmonary function. The following clinic-pathologic factors were included into analyses: age, gender, tumor location, FEV1, tumor differentiation, tumor histology, pathologic T stage, tumor subtype, STAS, pleural invasion and lymphovascular invasion.

### Statistical analyses

We use Kolmogorov–Smirnov test and Shapiro–Wilk test to evaluate the distribution for continuous variables. If the variables did not follow the normal distribution, we used the Mann–Whitney U Test to compare the median and the inner interquartile range (IQR) between the two groups. The linear regression model was used to determine the effects of surgical methods on resection margin. Univariable and multivariable analyses were performed. The variables that were significantly different between the two groups, as well as the variables that were associated with the resection margin, were selected into the maximum model. They were controlled and adjusted together to eliminate the effects of confounders. We used the coefficient *p*-values to decide whether to include variables in the final model. If there is a variable with a *p*-value greater than 0.05 in the multiple linear regression mode, it will be removed, and the variables that are still statistically significant are retained as the final mode.

All calculations were performed using the IBM SPSS Statistics for Windows, Version 22.0 (IBM Corp., Armonk, NY). Statistical analysis with a p value less than 0.05 was considered statistically significant.

## Results

**Fig. 2:**
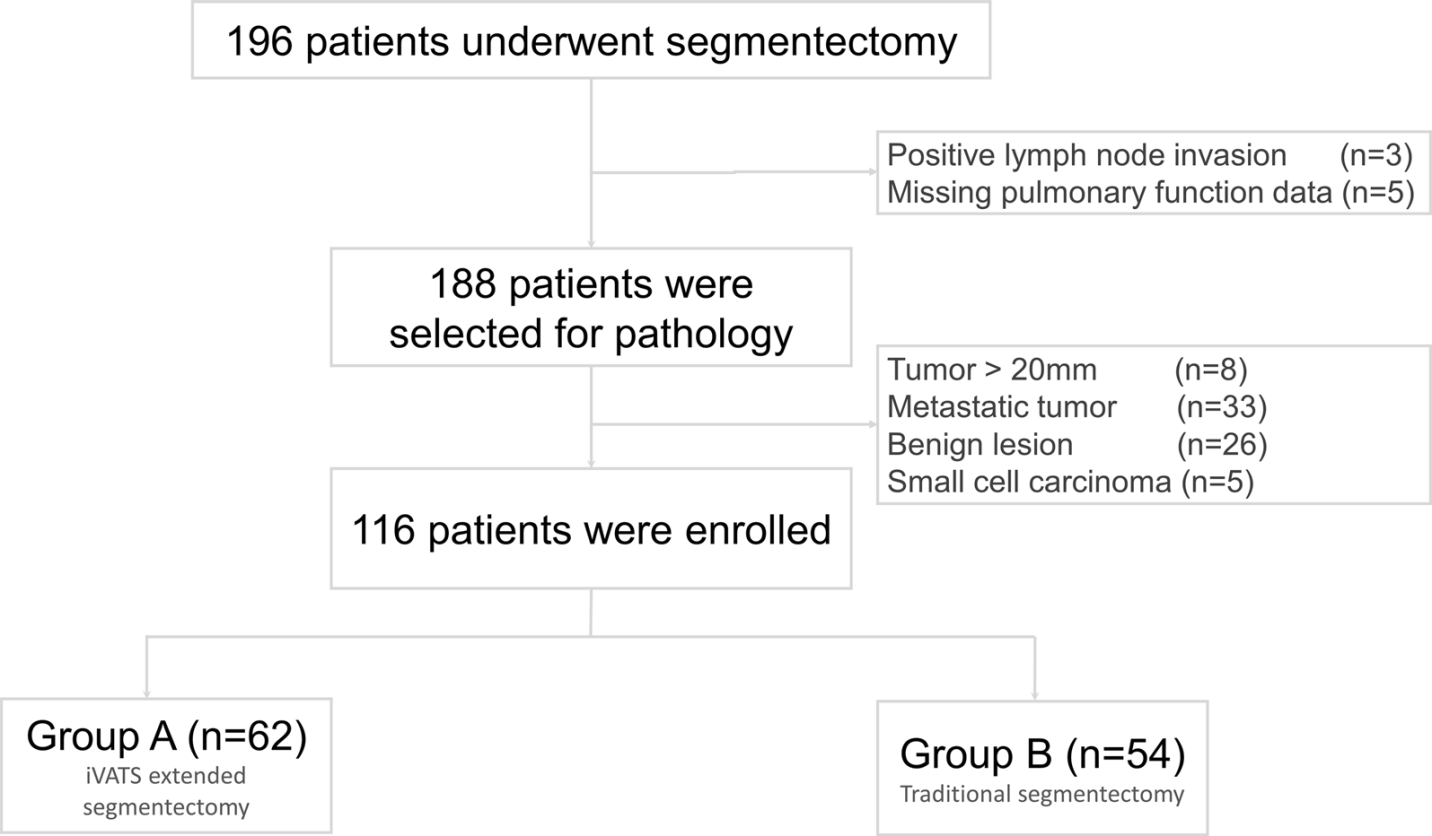
Patient enrollment process

In this study, 196 patients with pulmonary nodules received segmentectomy (Fig. 2). 80 patients were excluded in this study as followed: 8 patients with tumor large 2 cm in size; 33 patients with metastatic tumor; 26 patients with benign tumor; 5 patients with small cell carcinoma. Thus, 116 patients were included for evaluation. 62 patients underwent iVATS extended segmentectomy, and the other 54 patients underwent traditional segmentectomy.

The clinicopathological characteristics of all study patients are shown in Table 1. The mean age was similar in each group (57.97 years old in group A vs. 60.57 years old in group B, *p* = 0.232). Both groups were female predominant (58.06% in group A vs. 62.96% in group B, *p* = 0.591). Most of the nodules were located in the upper lobe, followed by the lower lobes and middle lobe. The FEV1 data showed no significant difference in each group (95.63% in group A vs. 89.14% in group B, *p* = 0.050).

**Table 1:** Clinical demographic data of patients

		iVATS extended (n = 62)		Traditional (n = 54)		*P*-value
		N	%	N	%	
Gender	Male	26	41.9	20	37.0	0.591
	Female	36	58.1	34	63.0	
Age	Median (IQR)	58.0	(52.0–64.0)	59.5	(54.0–67.0)	0.232
Tumor location	Right upper lobe	28	45.2	14	25.9	0.049
	Right middle lobe	1	1.6	1	1.9	
	Right lower lobe	10	16.1	10	18.5	
	Left upper lobe	18	29.0	15	27.8	
	Left lower lobe	5	8.1	14	25.9	
FEV1 (%)	Median (IQR)	93.1	(86.1–105.0)	89.4	(79.5–97.3)	0.050
Differentiation	Well	22	35.5	13	24.5	0.308
	Moderately	39	62.9	37	69.8	
	Poor	1	1.6	3	5.7	
Histology	Adenocarcinoma	62	100.0	49	90.7	0.020
	Squamous cell carcinoma	0	0.0	3	5.6	
	Others	0	0.0	2	3.7	
Pathologic T stage	Tis	12	19.4	5	9.3	0.076
	Tmi	14	22.6	7	13.0	
	T1	36	58.1	42	77.8	
Subtype	Lepidic	26	41.9	18	34.6	0.424
	Non-lepidic	36	58.1	34	65.4	
STAS	No	57	91.9	48	88.9	0.576
	Yes	5	8.1	6	11.1	
Pleural invasion	No	60	96.8	50	92.6	0.415
	Yes	2	3.2	4	7.4	
Lymph invasion	No	61	98.4	48	88.9	0.049
	Yes	1	1.6	6	11.1	
Enough margin	No	15	24.2	23	42.6	0.035
	Yes	47	75.8	31	57.4	

FEV1, forced expiratory volume in one second; STAS, spread through air spaces; Lymph invasion, lymphovascular invasion; IQR, interquartile range between the 25th percentile and the 75th percentile

Most of the NSCLC tumors were moderately differentiated (62.90% in group A vs. 69.81% in group B), followed by well differentiated and poorly differentiated. The predominant histology was adenocarcinoma (100% in group A vs. 90.74% in group B). T1 was the predominant pathologic T stage (58.06% in group A and 77.78% in group B). 22.58% in group A and 12.96% in group B revealed minimal invasive carcinoma. 19.35% in group A and 9.26% in group B showed adenocarcinoma in situ. The predominant subtype was non-lepidic (58.06% in group A vs. 65.38% in group B). In both groups, less than 15% showed STAS and less than 10% showed pleural invasion. Since most of the continuous variables in this study did not follow the normal distribution, we used the Mann–Whitney U Test to compare the median and the inner interquartile range (IQR) between the two groups. As for the categorical variables, we used the Chi-Squared Test or Fisher's Exact Test to compare the number and proportion between the two groups.

The outcomes of iVATS extended segmentectomy and traditional segmentectomy are presented in Table 2. As expected, the operative time (109.11 min vs. 112.80 min) and blood loss (60.08 ml vs. 65.93 ml) were similar in both groups. We use the total tumor size to evaluate in this study because over half of the nodule is ground-glass nodule (GGN). The mean diameter of nodules was also similar (10.26 mm in group A vs. 11.65 mm in group B, *p* = 0.071). The mean nodule depth was about 5 mm beneath the pleura in both groups (5.81 mm in group A vs. 4.72 mm in group B, *p* = 0.556).

**Table 2:** Outcomes of traditional segmentectomy and iVATS extended segmentectomy

	iVATS extended segmentectomy (n = 62)					Traditional segmentectomy (n = 54)					
Overall	Mean	SD	Median	Q_1_	Q_3_	Mean	SD	Median	Q_1_	Q_3_	*P*-value
OP time (min)	109.1	20.3	106.0	94.0	123.0	112.8	18.9	108.0	98.0	127.0	0.259
Blood loss (ml)	60.1	55.0	50.0	20.0	50.0	65.9	64.9	50.0	20.0	100.0	0.871
Nodule size (mm)	10.3	4.1	10.0	7.0	13.0	11.6	4.1	11.5	8.0	15.0	0.071
Distance to the pleura (mm)	5.8	6.1	3.0	1.0	10.0	4.7	4.8	3.0	1.0	6.0	0.556
Closest to staple line (mm)	17.9	9.3	15.0	10.0	25.0	14.1	8.8	12.0	8.0	20.0	0.037
Margin/tumor ratio	2.1	1.4	1.9	1.0	2.8	1.4	1.0	1.2	0.6	2.0	0.003

Q1 = percentile 25; Q3 = percentile 75; *P*-value by Mann–Whitney *U* Test

The resected segment are showed in Table 3. Most of the segmentectomy was done in segment RS1, RS6, LS1 + 2 and LS6. We can find that LS6 is much more in traditional segmentectomy than iVATS segmentectomy. The distribution of resected segment is significant different, the *p*-value is 0.003.

**Table 3:** Results of resected segment distribution

		iVATS extended (n = 62)		Traditional (n = 54)		*P*-value
		N	%	N	%	
Tumor location	RS1	12	19.4	7	13.0	0.003
	RS2	10	16.1	4	7.4	
	RS3	6	9.7	3	5.6	
	RS5	1	1.6	1	1.9	
	RS6	7	11.3	8	14.8	
	RS7+8	1	1.6	0	0	
	RS9+10	2	3.2	2	3.7	
	LS1+2 LS	10	16.1	9	16.7	
	LS3	5	8.1	4	7.4	
	LS4+5	3	4.8	2	3.7	
	LS6	4	6.4	11	20.4	
	LS8	0	0	1	1.9	
	LS9+10	1	1.6	2	3.7	

### Margin analysis and enough margin rate

We investigated the results of resection margin distance, M/T ratio and enough margin rate in both groups. There was a significant difference in resection margin to a staple line (17.94 mm in group A vs. 14.15 mm in group B, *p* = 0.037), and there was a significant difference in the M/T ratio (2.08 in group A vs. 1.39 in group B, *p* = 0.003). The enough margin rate was 75.81% (n = 47) in group A and 57.41% (n = 31) in group B.

**Fig. 3:**
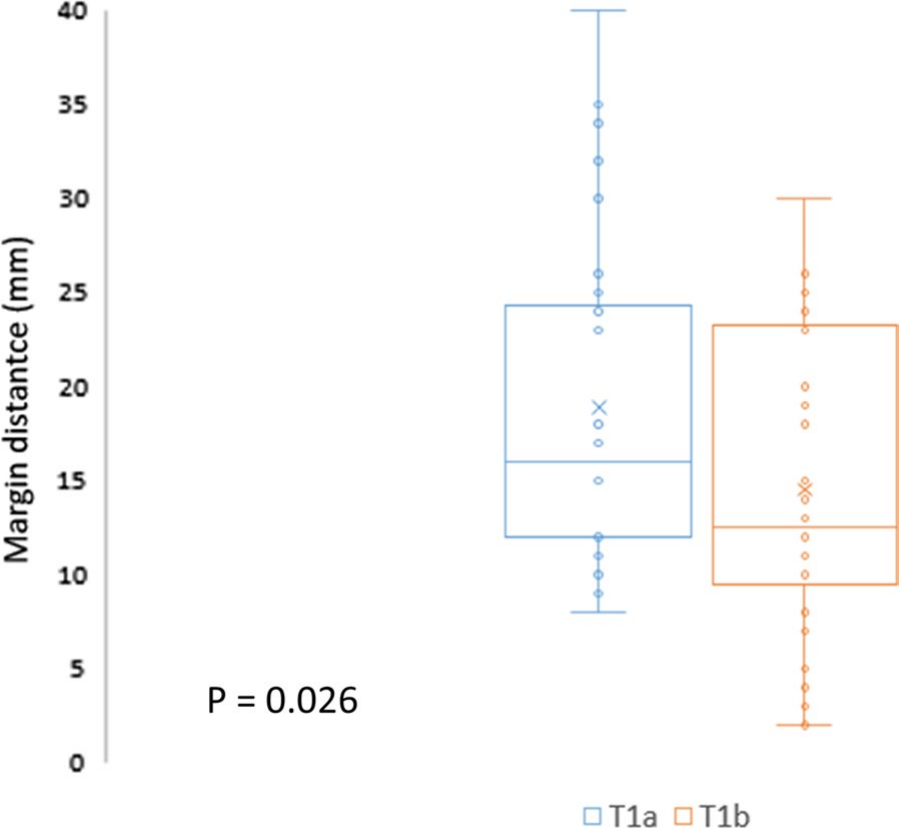
Subgroup analysis of iVATS extended segmentectomy, the T1a lesions had larger margin distances than did T1b lesions (19.85 mm vs. 14.83 mm, *p* = 0.026)

The subgroup analysis of iVATS extended segmentectomy appears in Fig. 3. We found that the T1a lesions had larger margin distances than did T1b lesions (19.85 mm vs. 14.83 mm, *p* = 0.026). We assumed that iVATS extended segmentectomy is more suitable for tumor lesions greater than 10 mm in diameter.

We performed additional wedge resection to create more resection margin if the margin was less than 5 mm. There are only 1 patient (1.6%) in iVATS extended segmentectomy group and 4 patients (7.4%) in traditional segmentectomy group required further wedge resection after we check margin at a back table.

Both univariable and multivariable linear regression models were analyzed (Table 4). In univariable analysis, only iVATS extended segmentectomy was found to be statistically associated with a larger resection margin. In multivariable analysis, iVATS extended segmentectomy, a tumor located in a lower lung lobe, and pathologic T1a were associated with a larger resection margin.

**Table 4:** Results of generalized linear model on resection margin

Parameter		Bivariable analysis (crude)				Multivariable analysis (adjusted)			
		β	SE	95% CI	*P*-value	β	SE	95% CI	*P*-value
Surgical type	iVATS extended segmentectomy	3.787	1.670	0.514 to 7.061	0.023	4.636	1.647	1.408 to 7.865	0.005
	Traditional segmentectomy (ref)	0.000				0.000			
Age		0.055	0.086	− 0.113 to 0.223	0.522	0.169	0.084	0.005 to 0.333	0.043
Location	Left lower lobe	2.994	2.485	− 1.877 to 7.865	0.228	6.132	2.399	1.431 to 10.833	0.011
	Left upper lobe	1.552	2.091	− 2.546 to 5.650	0.458	3.299	1.961	− 0.545 to 7.143	0.093
	Right lower lobe	3.860	2.442	− 0.927 to 8.646	0.114	5.568	2.307	1.047 to 10.090	0.016
	Right middle lobe	− 6.690	6.505	− 19.441 to 6.060	0.304	− 6.261	5.948	− 17.919 to 5.398	0.293
	Right upper lobe (ref)	0.000				0.000			
FEV1		0.037	0.052	− 0.065 to 0.138	0.481				
Histology	Others	− 14.541	6.383	− 27.051 to − 2.030	0.023	− 20.697	6.923	− 34.267 to − 7.128	0.003
	Squamous cell carcinoma	− 4.541	5.235	− 14.801 to 5.720	0.386	− 7.456	5.090	− 17.433 to 2.521	0.143
	Adenocarcinoma (ref)	0.000				0.000			
T stage	1b	− 2.090	1.696	− 5.413 to 1.234	0.218	− 3.429	1.662	− 6.687 to − 0.171	0.039
	1a (ref)	0.000				0.000			
Lymph invasion	Yes	0.121	3.576	− 6.887 to 7.128	0.973	7.977	3.982	0.173 to 15.782	0.045
	No (ref)	0.000				0.000			

β, regression coefficient; CI, confidence interval; Lymph invasion, lymphovascular invasion

## Discussion

Our study is a retrospective study investigating iVATS extended segmentectomy for patients with NSCLC, based on the 8th AJCC staging system [11]. Our previous study showed that the mean time from localization to skin incision was 23.57 min with iVATS [10]. The complication rate of iVATS is only 3.2%. Pneumothorax was noted after localization. However, the surgery was done immediately after localization with the iVATS technique. No further intervention was done. We suggested that iVATS extended segmentectomy can create more resection margin and make a precise resection.

The optimal resection margin was researched and it is believed that it was associated with lower tumor relapse and better overall survival [12]. Sawabata et al. suggested that margin distance greater than the maximum tumor diameter was considered to be the optimal margin [13]. Schuchert et al. reported that the recurrence rate was significantly higher if the resection margin was less than 2 cm and the M/T ratio was less than 1 [14]. Other researchers suggested that the optimal resection margin is 1.5 cm for NSCLC that is less than 2 cm in diameter [15]. In all, the resection margin must be at least 15 to 20 mm or larger than the tumor’s diameter to prevent tumor relapse for early-stage NSCLC.

Nowadays, the breakthrough of iVATS can provide easier pulmonary resection. The advanced intra-operative imaging-guided techniques make resection more precise [9]. Several studies have discussed in detail the experience of iVATS with cone-beam CT. Hsieh et al. reported the learning curve of iVATS technique is around 30 patients [16]. Chao et al. applied dual-marker localization technique with iVATS [17]. The bilateral lung nodules localization and resection was also reported [8]. The iVATS technique also decreases the risk of coil dislodge and dye fading. Because of these advantages, iVATS extended segmentectomy provides the ability to make more precise resection margins.

We also note that a tumor located in a lower lobe or less than 10 mm in diameter can be treated better with iVATS extended segmentectomy. A previous study reported that tumors located at the superior segment or left upper lobe may have lower local recurrence risk than other segments with extended segmentectomy [7]. Sienel et al. even emphasized that segmentectomy within the S1-3 region should be avoided whenever possible due to higher local recurrence [12]. The reason why our study showed that the bilateral lower lobes have more resection margin with iVATS extended segmentectomy may be due to the lower lobes being larger than the upper and middle lobes. There are more direction and width of extended resection that we can select. A T1a lesion can obtain about 5 mm more resection margin than a T1b lesion. Although the NCCN guidelines suggest that segmentectomy can be done for peripheral nodules less than 2 cm in diameter with ground-glass appearance larger than 50%, we recommend segmentectomy should only be performed when the nodule’s diameter is less than 1 cm. We do not even recommend iVATS extended segmentectomy for nodules larger than 1 cm in diameter.

It’s extremely important to define the pathological margin. The mean microscopic wedge resection margin distance was 11 mm smaller than the pleural surface-based margin [18]. We measured the pleural surface-based margin on a back table. Thus there is seldom patients with margin less than 5 mm and need additional wedge resection. The pathologist will further measure the microscopic margin distances. It’s 11 mm smaller than the pleural surface-based margin. This is why the enough margin rate was only 75.81% (n = 47) in iVATS extended segmentectomy group even we made 2 cm more wedge resection.

The long-term outcomes of lobectomy and segmentectomy have been controversial in recent decades. A recent meta-analysis study analyzed 28 studies about patients with stage I NSCLC. It claimed that lobectomy and segmentectomy reveal comparable outcomes when the tumor is less than 2 cm in diameter [19]. Most of the studies agreed that segmentectomy is preferred when the tumor is less than 1 cm in diameter. For tumors from 1.1 to 2.0 cm in diameter, lobectomy and segmentectomy could lead to equivalent survival rates [20]. Our study showed that we can obtain a resection margin of about 20 mm with iVATS extended segmentectomy when the tumor is less than 1 cm and a 15 mm resection margin when the tumor is between 1 and 2 cm. More observation periods are needed to compare the long-term outcomes of iVATS extended segmentectomy with those of traditional segmentectomy and lobectomy.

We believe that the additional subsegmentectomy may make better resection margin. However, it require 3D preoperative imaging to make nodule location more precise. The technique of additional subsegmentectomy is also more complicated than extended segmentectomy. The 3D preoperative imaging with iVATS procedure may be the trend in the future to help more dedicate segmentectomy decision.

This study is the first study to report on iVATS extended segmentectomy and to compare outcomes between iVATS extended segmentectomy and traditional segmentectomy. We demonstrated that iVATS extended segmentectomy is useful and makes extended segmentectomy more precise. However, the hybrid operating room with robotic C-arm cone beam CT has not been popularized. The iVATS technique needs time and promotion to become widespread. There are some limitations of our study. First, this is a retrospective study which may have selection bias, and this could affect the data analysis. The decision to perform either iVATS extended segmentectomy or traditional segmentectomy was rather subjective. The resection margin was randomly judged by the different pathologist in our institute. Those factor potentially lead to selection bias and affect the outcomes. Second, the observation period is not long enough. We need more observation periods to analyze how resection margin relates with tumor relapse and overall survival. Third, there was limited data about post-operative lung function. The preservation of lung function should also be analyzed in the two groups.

## Conclusions

iVATS extended segmentectomy can provide more resection margin than traditional segmentectomy. An iVATS extended segmentectomy is more suitable to perform when the nodule’s diameter is less than 10 mm.

## Data Availability

The datasets used and/or analyzed during the current study are available from the corresponding author on reasonable request.

## Abbreviations

NSCLC: Non-small cell lung cancer
iVATS: Image-guided video-assisted thoracoscopic surgery
NCCN: National Comprehensive Cancer Network
FEV1: Forced expiratory volume in one second
STAS: Spread through air spaces
M/T ratio: Margin/tumor diameter ratio
IQR: Inner interquartile range
